# Voneinander lernen, miteinander behandeln

**DOI:** 10.1007/s00105-021-04896-0

**Published:** 2021-10-22

**Authors:** Wolf-Henning Boehncke, Jan Ehrchen, Ulrich Mrowietz

**Affiliations:** 1 Service de Dermatologie et Vénéréologie, Hôpitaux Universitaires de Genève, Rue Gabrielle-Perret-Gentil 4, CH–1211 Genf, Schweiz; 2 Klinik für Hautkrankheiten, Universitätsklinikum Münster (UKM), Von-Esmarch Str. 58, 48149 Münster, Deutschland; 3 Psoriasis-Zentrum Klinik für Dermatologie, Universitätsklinikum Schleswig-Holstein, Campus Kiel, Arnold-Heller-Str. 3, Haus U27, 24105 Kiel, Deutschland

Viele verschiedene Erkrankungen können Hautsymptome verursachen, daher muss in der Dermatologie häufig über den Tellerrand des eigenen Fachgebietes hinaus gedacht werden. In den letzten Jahrzehnten hat sich gezeigt, dass in Bezug auf die Pathogenese und Therapie entzündlicher Erkrankungen sehr viele organunabhängige Grundprinzipien bestehen. Gerade im Bereich chronischer, entzündlicher Hauterkrankungen ist das interdisziplinäre Management in den letzten Jahren salonfähig geworden, an einigen universitären Standorten gibt es schon Zentren für Entzündungsmedizin. Diese können die notwendigen kollegialen Netzwerke ergänzen.

In dem Leitthema dieses Heftes wollen wir rheumatologische Aspekte unseres Fachgebietes diskutieren. Sehr deutlich wird die Nähe zwischen der Dermatologie und der Rheumatologie in der ambulanten, spezialfachärztlichen Versorgung (ASV) rheumatologischer Patienten. Hier ist die Dermatologie ein zwingender Bestandteil des von den Kostenträgern vorgeschriebenen interdisziplinären ASV-Kernteams.

Dies ist sinnvoll, da es eine Vielzahl rheumatologischer Erkrankungen gibt, die mit Hautsymptomen einhergehen. Typische Beispiele sind die klassischen Autoimmunerkrankungen wie der systemische Lupus erythematodes oder die Dermatomyositis, aber auch die aktuell vermehrt in den Fokus rückenden autoinflammatorischen Erkrankungen. Diese schwerwiegenden Erkrankungen sollten auch im allgemeinen dermatologischen Patientengut nicht übersehen werden, sie sind aber insgesamt selten.

In dem Leitthema dieser Ausgabe „Haut und Rheumatologie“ möchten wir uns aber für die tägliche Praxis auf die beiden häufigsten rheumatologischen Erkrankungen fokussieren:

In dem Beitrag von *Voigt et al*. werden typische Hautveränderungen bei der *rheumatoiden Arthritis* übersichtlich zusammengefasst. Wenn bei einem Patienten entsprechende Hautveränderungen und zusätzlich Gelenkbeschwerden bestehen, aber bisher keine rheumatologische Abklärung erfolgte, hat der behandelnde Dermatologe eine besondere Verantwortung. Da durch eine frühe Therapie der rheumatoiden Arthritis (gemäß dem rheumatologischen Therapiekonzept „*hit hard and early“*) irreversible Gelenkdestruktionen verhindert werden können, sollte in der dermatologischen Praxis bei entsprechenden Hautveränderungen immer gezielt nach Gelenkbeschwerden gefragt werden. Im Idealfall leitet der Dermatologe dann auch die Diagnostik ein (z. B. Bestimmung der Antikörper gegen citrullinierte Proteine) und vermittelt im Folgenden eine rheumatologische Vorstellung. Hier kann ein ärztliches Telefonat den betroffenen Patienten häufig viele Monate Wartezeit ersparen.

Bei der *Psoriasisarthritis* kommt es hingegen nicht nur darauf an, die Erkrankung in der dermatologischen Praxis zu erkennen, vielmehr sollte auch die Therapie dieser Erkrankung in enger interdisziplinärer Absprache erfolgen. In dem Beitrag des Kollegen *Boehncke* werden daher nicht nur die Klinik und Diagnostik, sondern auch therapeutische Aspekte der Psoriasisarthritis praxisnah dargestellt. Bei einem entsprechenden Verdacht sollte bereits in der dermatologischen Praxis eine Therapie eingeleitet werden, die sowohl auf die Psoriasis als auch auf die Gelenkmanifestation wirkt. Im Idealfall erfolgt bezüglich der Therapie ein direkter ärztlicher Kontakt zwischen den Dermatologen und Rheumatologen. Dies ist umso einfacher, desto besser man sich kennt. Daher ist es sehr erfreulich, dass zunehmend interdisziplinäre Fortbildungen angeboten werden, die jetzt auch wieder als Präsenzveranstaltungen möglich sind. Trotz der unbestreitbaren Vorteile der Videokonferenzen lässt sich zumindest für das Aufbauen kollegialer Netzwerke der direkte Kontakt schwer ersetzen.

